# Ivalin Induces Mitochondria-Mediated Apoptosis Associated with the NF-κB Activation in Human Hepatocellular Carcinoma SMMC-7721 Cells

**DOI:** 10.3390/molecules24203809

**Published:** 2019-10-22

**Authors:** Zhuo Han, Fang-yuan Liu, Shi-qi Lin, Cai-yun Zhang, Jia-hui Ma, Chao Guo, Fu-juan Jia, Qian Zhang, Wei-dong Xie, Xia Li

**Affiliations:** 1 Marine college, Shandong University, Weihai 264209, China; hanzhuo1013@gmail.com (Z.H.); super70732635@163.com (C.G.); zhangqianzq@sdu.edu.cn (Q.Z.);; 2 School of Pharmaceutical Sciences, Shandong University, Jinan 250012, China

**Keywords:** Ivalin, *Carpesium divaricatum*, hepatocellular carcinoma, mitochondria-mediated apoptosis, NF-κB

## Abstract

Ivalin, a natural compound isolated from *Carpesium divaricatum*, showed excellent microtubule depolymerization activities among human hepatocellular carcinoma in our previous work. Here, we investigated its functions on mitochondria-mediated apoptosis in hepatocellular carcinoma SMMC-7721 cells. DAPI (4′,6-diamidino-2-phenylindole) staining, annexin V-fluorexcein isothiocyanate (FITC) apoptosis detection, and western blotting were applied to explore the apoptotic effect of Ivalin. Next, the induction effect of Ivalin on the mitochondrial pathway was also confirmed via a series of phenomena including the damage of mitochondria membrane potential, mitochondria cytochrome c escape, cleaved caspase-3 induction, and the reactive oxygen species generation. In this connection, we understood that Ivalin induced apoptosis through the mitochondrial pathway and the overload of reactive oxygen species. Furthermore, we found that the activation of nuclear factor-κB (NF-κB) and subsequent p53 induction were associated with the apoptotic effect of Ivalin. These data confirmed that Ivalin might be a promising pro-apoptotic compound that can be utilized as a potential drug for clinical treatment.

## 1. Introduction

Clinical data have shown that hepatocellular carcinoma (HCC) is the most common and dominating primary tumor with a high incidence rate in adults worldwide [1,2,3]. Since many advanced treatments have been used in recent years, chemotherapy is still widely applied to treat HCC in the research of clinical trials [4]. However, both the occurrence of drug resistance and recurrences are the key hurdles associated with chemotherapy [5]. Therefore, the exploration and evolution of novel therapeutic agents for the treatment of HCC are greatly of need.

**Figure 1:**
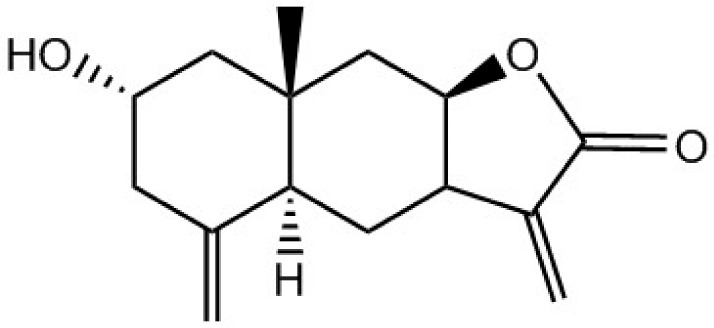
Structure of Ivalin.

Abstracted compounds from plants utilized into an effective anticancer drug is one of the fastest developing therapeutics for chemotherapy [6,7]. The large family Asteraceae contains over 25,000 species (such as *Aster* and *Carpesium*) and many species have shown that it is best utilized as sources of edible oils, vegetables, pesticides, medicines and so on. In addition, it is considered to be an ideal source of effective natural compounds with the structure of sesquiterpenoids, particularly, an eudesmane framework [8]. Eudesmane-type sesquiterpenoids and their biological functions including antifungal, antitumor, and antibacterial have been the fastest growing field of pharmacological and synthetic studies during the last two decades [9]. Telekin and 1-oxoeudesm-11(13)-eno-12,8a-lactone (OEL), examples of compounds with an eudesmane framework, have been reported to strongly restrain cell proliferation via the induction of mitochondria-mediated apoptosis [10,11]. Ivalin (Figure 1), another compound with an eudesmane framework, was abstracted from the traditional herb *Carpesium divaricatum* [12]. Our previous works demonstrated that Ivalin can serve as a novel microtubule inhibitor by depolymerizing microtubules and resulted in cell proliferation inhibition in hepatocellular carcinoma SMMC-7721 cells [13]. Here, we report that Ivalin enforced the procedure of apoptosis in the same cells by triggering reactive oxygen species (ROS) generation and the participation of the mitochondria pathway.

Nuclear factor-κB (NF-κB), a conventional transcription factor, is important for the execution and control of apoptosis [14]. NF-κB, in different given stimulations such as in ROS accumulation and cancer therapeutic agents, complexly regulates the program of apoptosis via its downstream target genes, which include the p53 protein and so on [15,16]. Detecting both the protein and mRNA levels of NF-κB and p53 will help us to further perceive and recognize the influence of pro-apoptosis and underlying theory of Ivalin. The results from these experiments showed that the initiate of NF-κB and subsequent p53 induction were associated with the apoptotic effect of Ivalin in SMMC-7721 cells.

## 2. Results

### 2.1. Apoptotic Effect of Ivalin

**Figure 2:**
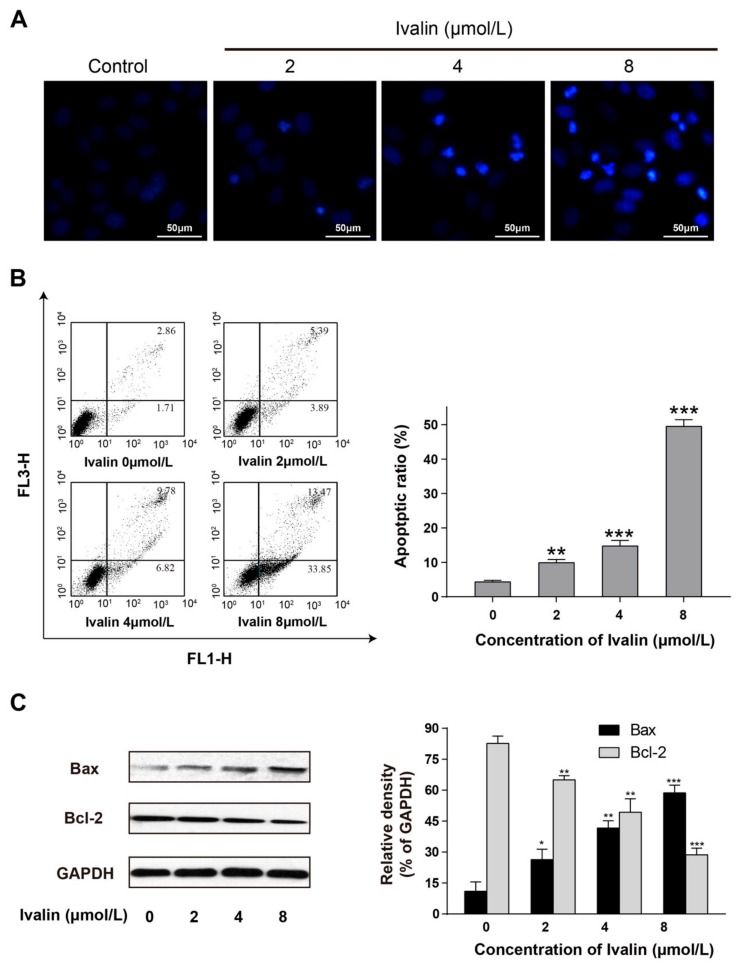
SMMC-7721 cells treated with Ivalin causing apoptosis. (**A**) Fluorescence micrographs of untreated and Ivalin treated SMMC-7721 cells with 4′,6-diamidino-2-phenylindole (DAPI). Magnification: 100×. (**B**) Results from the flow cytometry analysis, the quantification of the apoptotic cells after indicate treatment. (**C**) Western blot showed that Ivalin induced apoptosis by enhancing the Bax and declining the Bcl-2 expression. * *p* < 0.05; ** *p* < 0.01, *** *p* < 0.001 vs. the control group.

Our previous studies confirmed that Ivalin (Figure 1) was significantly cytotoxic to SMMC-7721 cells (IC50: 4.34 ± 0.10) with a lower effect toward the normal cell line HL7702 (IC50: 25.86 ± 0.87) [13]. In response to characterizing the cell growth inhibition effect of Ivalin, we monitored morphological changes in SMMC-7721 cells after 24 h of treatment. Compared to the untreated cells, Ivalin treatment increased the apoptotic body formation as well as nuclear condensation, which were the significant morphologic alterations related to apoptosis (Figure 2A).

When cells were undergoing apoptosis, the phosphatidylserine in the inter surface of the plasma membrane transforms to the outer surface, which can be stained with Annexin V. In this connection, we performed flow cytometry to further quantify the apoptotic effect of Ivalin via dual stained cells with Annexin V-fluorexcein isothiocyanate and propidium iodide. The results shown in Figure 2B revealed that the proportion of Annexin V-stained cells increased with the percentages increased from 4.57%, 9.28%, 16.6%, to 47.32% after treating with 0 to 8 μmol/L Ivalin, respectively. Therefore, we believe that Ivalin may strongly increase the ratio of apoptotic cells in SMMC-7721 cells.

The Bcl-2 family consists of members with a pro-apoptotic or the opposite effect and the balance between them may regulate the fate of cells [17,18]. Bcl-2 and Bax, the most common proteins with vital roles in the Bcl-2 family, were analyzed by western blot after Ivalin treatment. Results revealed that Ivalin-treatmen triggered the altered expression of Bcl-2 and Bax in SMMC-7721 cells (Figure 2C). The increase in the Bax protein and decrease in the Bcl-2 protein expression levels further confirmed the pro-apoptotic effect of Ivalin as suggested above.

### 2.2. Ivalin Triggered the Loss of Mitochondrial Membrane Potential (MMP) in SMMC-7721 Cells

**Figure 3:**
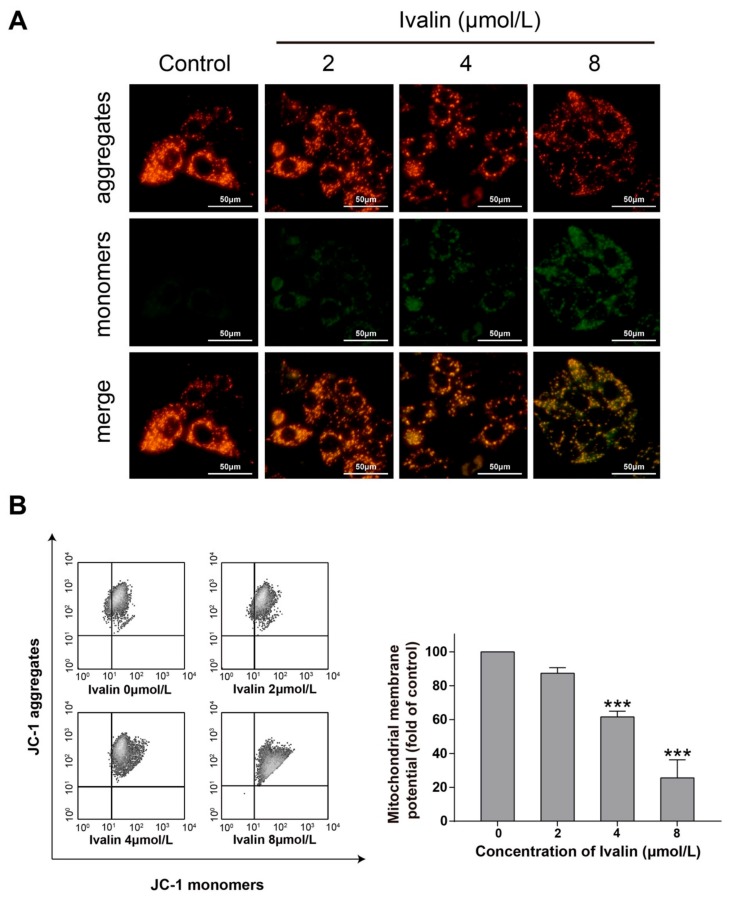
Effects of MMP generation in Ivalin-treated cells. (**A**,**B**) After Ivalin treatment for 24 h, flow cytometry and fluorescence microscope were used to detect cellular mitochondrial membrane potential. (**A**) Ivalin treatment decreased the red fluorescence intensity (aggregates) and increased green fluorescence intensity (monomers) in SMMC-7721 cells, indicating that Ivalin reduced the mitochondrial membrane potential, thereby leading to mitochondrial dysfunction. (**B**) Ivalin induced the loss of mitochondrial membrane potential as shown by flow cytometry. *** *p* < 0.01 vs. the control group.

**Figure 4:**
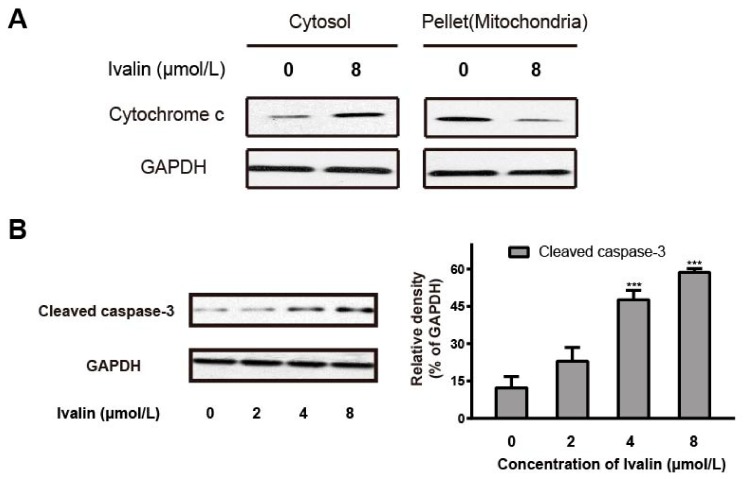
Ivalin trigged apoptosis by means of the mitochondria activation. (**A**) The cytochrome c in mitochondria with the stimulation of Ivalin inflowed into the cytosol. (**B**) Cleaved caspase-3 was increased with the treatment of Ivalin. *** *p* < 0.001 vs. the control group.

We next stained the cells with JC-1 to measure the cellular MMP in response to Ivalin treatment. Cells treated with Ivalin led to the loss of MMP in a concentration-dependent manner (Figure 3). Meanwhile, the increased mitochondrial membrane permeability in treated cells may result in the translocation of mitochondria cytochrome c to cytosol. Figure 4A illustrates an apparent release of cytochrome c from the mitochondria to cytosol in the experimental groups. Furthermore, the treatment with Ivalin concentration-dependent increased the level of cleaved caspase-3 in the experimental groups (Figure 4B). The above findings indicate that the mitochondria-mediated pathway was associated with Ivalin-induced apoptosis.

### 2.3. The Generation of ROS Appeared in Ivalin-Treated Cells

**Figure 5:**
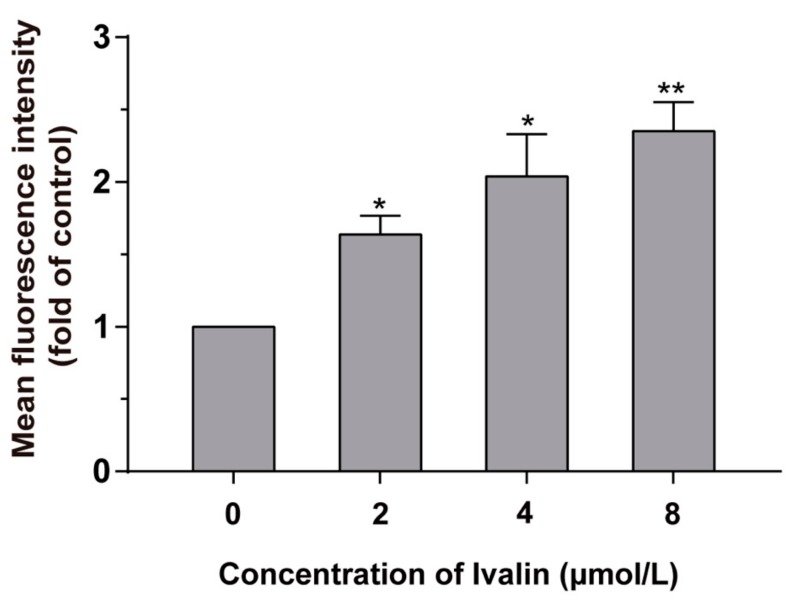
Ivalin induced the generation of intracellular ROS. Graph shows the fluorescence intensities of DCF in the SMMC-7721 cells exposed to Ivalin in contrast to control. The fluorescence intensity of control group was set as 1. * *p* < 0.05; ** *p* < 0.01 vs. the control group.

Intracellular ROS generation always appears during the process of mitochondrial obstruction. Therefore, we detected the fluorescence produced by dichlorofluorescein (DCF) by evaluating the possession of ROS by the flow cytometric assay to determine whether Ivalin can induce the accumulation of ROS. The group of treated and untreated cells were incubated with DCF-DA for 30 min and analyzed via flow cytometry. The results are shown in Figure 5 with a greater generation of ROS in the experimental groups in contrast to the untreated group.

### 2.4. Ivalin Modulated the Protein and mRNA Levels of NF-κB, IκB, and p53

**Figure 6:**
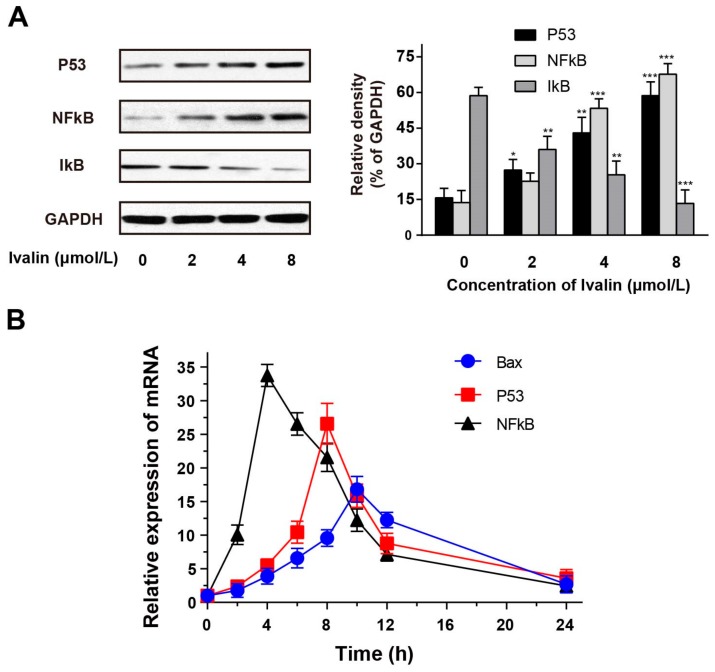
Trends of Ivalin on the p53, NF-κB and IκB expression. (**A**) The expressions of relative proteins were measured by western blotting. * *p* < 0.05; ** *p* < 0.01, *** *p* < 0.001 vs. the control group. (**B**) Relative mRNA expression values were calculated by real-time PCR. The quantity of each mRNA was relative to the glyceraldehyde-3-phosphate dehydrogenase (GAPDH) mRNA levels.

Currently, a large member of anticancer compounds take effect in coping with hazardous substances by targeting transcription activators like nuclear factor-κB (NF-κB) and p53 regulating ROS [19,20,21]. In order to further perceive and recognize the role of Ivalin in apoptotic induction, we experimented with the expression of NF-κB, IκB, and p53 through western blot analysis. Ivalin-treatment enhanced the expressions of NF-κB and p53, but decreased that of IκB protein (Figure 6A).

We also performed real-time PCR to evaluate if there had been any alteration in the NF-κB, p53, and Bax mRNA levels in the presence of Ivalin treatment. The data revealed that Ivalin time-dependently induced the gene expressions of NF-κB, p53, and Bax (Figure 6B). NF-κB was activated before p53, and the activation of NF-κB mRNA reached the highest levels early, about 4 h. Therefore, the effective activation of NF-κB was associated with the apoptotic effect of Ivalin.

## 3. Discussion

In our previous work, we reported the ideal microtubule depolymerization activities of Ivalin in SMMC-7721 cells [13]. In this study, we found that Ivalin treatment may lead to obviously apoptotic features including apoptotic body formation and nuclear condensation in the same cells. Furthermore, in the presence of an indicated concentration of Ivalin, a significant increase in the proportion of apoptotic cells was observed through double staining by annexin V-FITC and PI. In this connection, we presumed the apoptotic effect of Ivalin in SMMC-7721 cells.

Apoptosis can be induced by the extrinsic (death receptor pathway) and intrinsic pathways (mitochondria-mediated pathway). During the mitochondria-mediated pathway, the increase in mitochondria membrane permeability led to the subsequent release of mitochondria cytochrome c to cytosol, activated the caspase-9 and its downstream effector caspases including caspase-3, and finally induced apoptosis [22]. The Bcl-2 family proteins such as Bax and Bcl-2 mediate the intrinsic pathway by changing the mitochondria outer membrane permeabilization [23]. In particular, a common gateway for the mitochondria-mediated apoptotic pathway is the required level of Bax protein [24]. Ivalin treatment resulted in inducing the expression of Bax and attenuating that of Bcl-2 protein in SMMC-7721 cells. Additionally, treatment with Ivalin also caused the increase in the mitochondria membrane permeability, which was confirmed by the loss of MMP and an obvious release of cytochrome *c* from mitochondria into cytosol. Furthermore, the induction of cleaved caspase-3 expression in Ivalin-treated cells was also observed in the experiment. All of these results confirmed the effective mitochondria-mediated apoptotic effect of Ivalin among SMMC-7721 cells.

NF-κB, a transcription factor first identified in 1986, provides an effective goal for the progress of inflammation and apoptosis [25]. Nowadays, more and more evidence suggests the positive role of NF-κB in apoptosis, while the negative role of it in the apoptosis process has been discussed for many years [26]. In fact, the function of NF-κB in mediating apoptosis strongly relies on the type of cancer, the stimulation, and the related subunit [27,28,29,30]. When under non-stimulated status, NF-κB is in a complex formed with IκB in the cytoplasm without activation. Extracellular stimuli bring about rapid phosphorylation of IκB, which will be further degraded by ubiquitinase or protease [31]. When the degradation of IκB is finished, NF-κB activates rapidly and subsequently translocates into the nucleus to react with the downstream targets [32,33,34]. A key example was a report that confirmed the important role of NF-κB in the induction of wild type p53 expression to initiate pro-apoptotic signaling in response to ROS accumulation [21].

Here, Ivalin treatment led to a greater generation of ROS in SMMC-7721 cells. In this connection, further investigation was performed to confirm whether NF-κB was involved in the mitochondria-mediated apoptotic effect of Ivalin. Western blot analysis revealed that Ivalin increased the expression of NF-κB and p53, but decreased the expression of IκB. Moreover, rt-PCR revealed that the mRNA levels of NF-κB, p53, and Bax increased after Ivalin treatment in a time-dependent manner. NF-κB was activated first, followed by the activation of p53 and Bax, and the expression of NF-κB mRNA reached the highest levels as early as about 4 h. This finding indicates that the NF-κB signaling pathway is involved in Ivalin-induced mitochondria-mediated apoptosis in SMMC-7721 cells.

To sum up, this study is the first to report that Ivalin induced mitochondria-mediated apoptosis associated with the NF-κB activation in SMMC-7721 cells. Ivalin treatment resulted in a significant generation of ROS in SMMC-7721 cells. In response to this, NF-κB was activated and served as a transcription factor to induce p53 activation, which subsequently induced Bax while decreasing Bcl-2 protein expression, which eventually led to mitochondria-mediated apoptosis in SMMC-7721 cells. However, the detail mechanisms responsible for the pro-apoptotic activity of Ivalin need to be explored further. Hence, Ivalin deserves further research to develop it into a promising chem-therapeutic agent or leading compound for anti-cancer agent searching.

## 4. Materials and Methods

### 4.1. Chemicals and Reagents

Ivalin (>98%), provided by Dr. Xie (Shandong University, Weihai, China) [12], was dissolved and its concentration adjusted by dimethylsulfoxide (DMSO) when required. DAPI (4′,6-diamidino-2-phenylindole) was acquired from Sigma-Aldrich Corp. (St. Louis, MO, USA). An Annexin V-fluorexcein isothiocyanate (FITC) Apoptosis Detection Kit was purchased from BD Biosciences (San Jose, CA, USA). Caspase-3, cytochrome c, p53, and NF-κB antibodies were obtained from Cell Signaling Technology (CST, Inc, Beverly, MA, USA). Bcl-2, Bax, and GAPDH antibodies were obtained from Abcam Inc. (Cambridge, MA, USA). Beyotime Institute of Biotechnology (Shanghai, China) supplied the cell mitochondria/cytosol isolation kit, JC-1 and DCFH-DA, to us.

### 4.2. Human Hepatocellular Carcinoma and Cell Culture

Shanghai Institute for Biological Sciences (SIBS) of Chinese Academy of Sciences (Shanghai, China) supplied the SMMC-7721 cell line (human hepatocellular carcinoma cell line) and we cultured the cells according to the supplier’s instructions.

### 4.3. DAPI Staining

DAPI staining assay, as previously described [35], was used to observe the change in the nucleus after Ivalin treatment.

### 4.4. Mitochondrial Membrane Potential

We performed JC-1 staining to measure the changes in mitochondrial membrane potential (ΔΨm) in SMMC-7721 cells after Ivalin treatment. Cells were treated with Ivalin (0 μM to 8 μM) for 24 h and dealt with JC-1 in the light of the instructions from the manufacturer. Stained cells were collected for flow cytometric analysis or fluorescence microscope observation. The results contained three independent experiments.

### 4.5. Apoptosis Detection

The apoptosis rate of SMMC-7721 cells after Ivalin 24 h treatment was evaluated by Annexin V-FITC and Propidium Iodide (PI) double staining, according to our previously described study [13].

### 4.6. Measurement of Intracellular ROS Levels

Cells were treated with Ivalin, as described above, for 24 h. The working principle of the kit was that with an increase in ROS, DCFH-DA can transform into DCFH, which reacts with ROS, presenting the fluorescence property. We took advantage of this to measure the change of intracellular ROS content via flow cytometry as previously described [35].

### 4.7. Western Blot Analysis

Western blot analysis, performed as previously described [36], was used to detect the expression levels of indicated protein. In the assay for cytochrome c measurement, we used a mitochondria/cytosol isolation kit to separate the mitochondrial proteins from cytosol proteins.

### 4.8. Real-Time PCR Analysis

Real-time PCR assay, as described in our previous work [36], was used to detect the mRNA levels of the NF-κB, p53, and Bax genes. We designed all needed primers using primer premier 5 and synthesis by Sangon Biotech Co Ltd. (Shanghai, China) for the NF-κB gene (sense primer: 5′-TAGAAACAGACCGAGGAG-3′ and anti-sense primer: 5′-ACTGGCTAATAAAGTGAATG-3′), p53 gene (sense primer: 5′-GTTTCCGTCTGGGCTTCT-3′ and anti-sense primer: 5′-CCTCAGGCGGCTCATAG-3′), and Bax gene (sense primer: 5′-TCAACTGGGGCCGGGTTGTC-3′ and anti-sense primer: 5′-CCTGGTCTTGGATCCAGCC-3′).

### 4.9. Statistical Analysis

All data are presented as mean ± SD (standard deviation) if appropriate. A comparison between two groups was performed with the Student’s *t*-test. * *p* < 0.05; ** *p* < 0.01, *** *p* < 0.001 vs. the control group.
